# Corrosive Sublimate as a Disinfectant against the Staphylococcus Pyogenes Aureus

**Published:** 1891-12

**Authors:** W. Schleppegrel

**Affiliations:** New Orleans


ARTICLE V.

### CORROSIVE SUBLIMATE AS A DISINFECTANT AGAINST THE STAPHYLOCOCCUS PYOGENES AUREUS.

A. C. Abbott (*Hopkins Hospital Bulletin*, No. 12, April, 1891) publishes the results of tests made upon cultures of the staphylococcus pyogenes aureus with a 1:

1000 solution of corrosive sublimate. Abbott finds that the disinfectant power of corrosive sublimate in the above concentration, when tested by methods which exclude the carrying over of minute quantities of the disinfectant, is not so great as has been claimed. He holds that in many of the experiments heretofore made to test corrosive sublimate the latter has been assigned a higher rank than it deserves, because some of it has been transferred to the culture medium, and has inhibited, but not destroyed, the growth.

His experiments were made upon liquid cultures containing sterilized sand. With cultures of this sort he was able, by filtration, to get a better distribution of the organisms in the liquid, avoiding microscopic clumps, which might interfere with the action of the disinfectant upon the organisms in the centre. Suspensions in water were also used, also filtered. Fresh cultures and fresh solutions of corrosive sublimate were used, of course, in every case.

Abbott finds that the number of organisms makes a difference in the efficacy of the disinfectant. The greater the number of organisms the more difficult the disinfection. Cultures vary in their resisting power,—organisms from one culture resisting better than those from another. Cultures in beef-tea resist better than suspensions in water. Organisms which remain alive after the action of the disinfectant are retarded in their growth and are weakened in virulence. Corrosive sublimate, in the proportion of 1 : 400,000, retarded growth in cultures of boullion containing peptone; 1 : 600,000 with peptone. The staphylococci, which have been attenuated by the action of the sublimate, regain their virulence when cultivated for some time on ordinary culture media.

TURPENTINE AS A GERMICIDE AND ANTISEPTIC.—Although the oil of turpentine (*oleum terebintkinæ*, U.S.P.) is not unknown as an antiseptic and germicide, its insolubility in water and irritating properties have hitherto made its use impracticable. That it has its special uses, however, in this connection, I have had abundant testimony.

It is a well-known fact among naturalists that, if the air of a cabinet be impregnated with the vapor of turpentine, the specimens are safe from the ravages of moths and like intruders so long as this condition of the air of the cabinet remains.

Having learned the advantage of turpentine in preserving entomological specimens, I concluded to try its germicidal properties in cases containing surgical instruments. A bacteriological examination made four weeks afterwards, and compared with the examination of cases not provided with turpentine, convinced me of its efficacy, and I soon afterwards applied the same principle to drawers containing towels, gauze, bandages, &c.

The method is simple. The turpentine is placed in flat, large-mouthed bottles at the bottom of each case or drawer, the volatility of the turpentine causing the vapor to impregnate the surrounding air.

Of late I have also placed my surgical instruments the night preceding an operation, in a flat dish containing oil of turpentine. The instruments are completely sterilized, are not injured by the submersion and are easily dried by a piece of sterilized gauze or towel. The characteristic odor of turpentine can be removed by ether.

The cheapness of turpentine, and the ease with which it may always be obtained, added to its special adaptability in preserving the aseptic condition of instruments,bandages, etc., by its vapor, may make it a valuable addition to the list of antiseptics and germicides.

I have also used benzol in the above manner. Its greater volatility gives it a more rapid germicidal action than turpentine, but its great inflammability admonishes caution in its use.—W. SCHLEPPEGREL, A. M., M. D., New Orleans, in *Medical News*.

